# Estimation of Phytoplankton Responses to Hurricane Gonu over the Arabian Sea Based on Ocean Color Data

**DOI:** 10.3390/s8084878

**Published:** 2008-08-21

**Authors:** Dongxiao Wang, Hui Zhao

**Affiliations:** Key Laboratory of Tropical Marine Environmental Dynamics, South China Sea Institute of Oceanology, Chinese Academy of Sciences, Guangzhou 510301, China

**Keywords:** Hurricane, phytoplankton blooms, Chlorophyll-*a* concentrations, Arabian Sea, remote sensing

## Abstract

In this study the authors investigated phytoplankton variations in the Arabian Sea associated with Hurricane Gonu using remote-sensing data of chlorophyll-*a* (Chl-*a*), sea surface temperature (SST) and winds. Additional data sets used for the study included the hurricane and Conductivity-Temperature-Depth data. Hurricane Gonu, presenting extremely powerful wind intensity, originated over the central Arabian Sea (near 67.7°E, 15.1°N) on June 2, 2007; it traveled along a northwestward direction and made landfall in Iran around June 7. Before Hurricane Gonu, Chl-*a* data indicated relatively low phytoplankton biomass (0.05-0.2 mg m^-3^), along with generally high SST (>28.5 °C) and weak wind (<10 m s^-1^) in the Arabian Sea. Shortly after Gonu's passage, two phytoplankton blooms were observed northeast of Oman (Chl-*a* of 3.5 mg m^-3^) and in the eastern central Arabian Sea (Chl-*a* of 0.4 mg m^-3^), with up to 10-fold increase in surface Chl-*a* concentrations, respectively. The Chl-*a* in the two post-hurricane blooms were 46% and 42% larger than those in June of other years, respectively. The two blooms may be attributed to the storm-induced nutrient uptake, since hurricane can influence intensively both dynamical and biological processes through vertical mixing and Ekman Pumping.

## 1. Introduction

Hurricane Gonu was a Category 5 tropical storm, according to the Saffir-Simpson scale, which occurred over the northern Arabian Sea during June 2007. This was an unprecedented event because no cyclones of this strength had ever reached the Gulf of Oman before. It was reported that the cyclone caused about $4 billion in damage and over 50 deaths in Oman alone, where Hurricane Gonu was considered the nation's worst natural disaster in 2007. Hurricanes and typhoons are violent weather events originated over tropical oceans, which may cause catastrophic results when passing over land or ports. They can also impact marine ecosystem. Phytoplankton blooms and increasing primary productions triggered by hurricanes and typhoons have been reported and attributed to nutrient-rich water supply from beneath the euphotic zones through enhanced ocean mixing and upwelling [1-5]. Due to strong wind stress curls and large wind speeds during their passages, tropical storms can produce strong upwelling, inertial resonance, and deep mixed layer, which induce phytoplankton blooms and other significant ocean responses [3-7]. Recent studies showed that hurricanes can also induce re-suspension of sediments through entrainment and upwelling as well as complex dynamic interactions such as excitation of internal waves or hurricane-mesoscale feedbacks in the eastern Gulf of Mexico and its coastal regions [3, 5].

Due to transient and violent characteristics of tropical storms, ship-borne observations are difficult and dangerous. Satellite remote sensing thus provides an effective means to measure cyclone-induced phytoplankton variations over a large domain at frequent intervals. Although good-quality chlorophyll-a (Chl-a) images are hard to obtain during a hurricane because the remote sensor is typically obscured by heavy clouds and fogs, it is possible to obtain some useful ocean-color images from Merged Aqua/SeaWiFS shortly after a hurricane.

**Figure 1.**
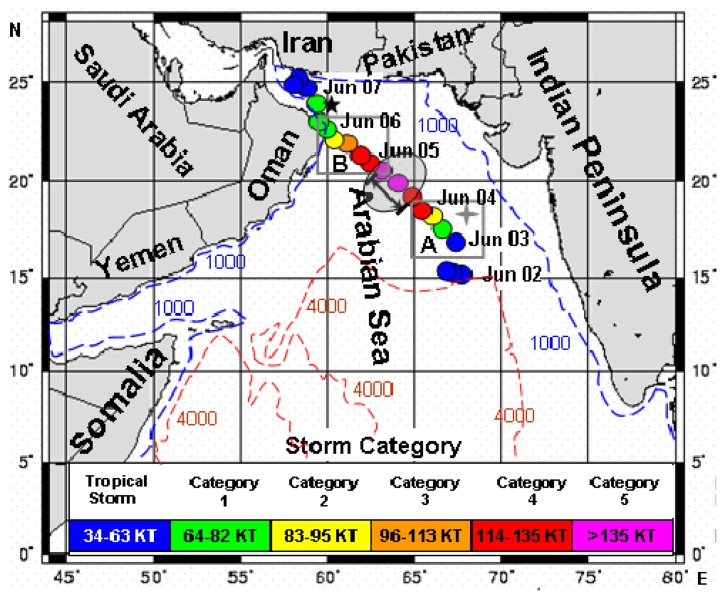
Bathymetry of the Arabian Sea, the maximum sustained wind (MSW) speeds and the track of Hurricane Gonu. Locations of Gonu every 6 hours were given by colored circles, where the colors indicated the MSW speeds (1 KT = 1 knot = 0.514 m s^-1^). Box A (16-19°N, 65-69°E) and Box B (20.5-23.5°N, 59.5-63.5°E) were used for area-averaged time series of May 15-July 15, 2007, where low SST, strong wind, and strong Ekman pumping velocity occurred during or after Gonu; the grey oval region marked by the double-headed arrow is the location with the strongest intensity of Gonu along its track.

The northern Arabian Sea (Figure 1) with an average depth of 2,700 m is strongly influenced by the seasonally reversed Indian monsoon system, which results in large seasonal variations in the upper-ocean circulation, sea surface temperature (SST), mesoscale eddies, mixed layer depth and heat budget [8-9]. Most of the offshore upper Arabian Sea corresponds to oligotrophic/mesotrophic tropical regions with low phytoplankton biomass [10]. In summer, strong southwestly (SW) winds blow across the Arabian Sea; in winter, the northeastly (NE) winds prevail. During the SW monsoon season, coastal upwelling occurs near the coast in the northwest Arabian Sea and open-ocean upwelling occurs off Oman and Somali [8-11]. The NE winds produce sea surface cooling and convective overturning conditions [11-14]. Both the SW monsoonal upwelling and the NE monsoonal convective overturning can cause significant regulation in primary productivity and zooplankton productivity through coastal upwelling and mesoscale eddies as well as mixing/turbulent entrainment in the Arabian Sea [15-22]. From SeaWiFS observations processed with the spectral-matching algorithm, summer phytoplankton blooms in the north Arabian Sea was better investigated during the prevalent period of dust storms associated with SW monsoon for the year 2000 [23]. Smith and Madhupratap investigated also spatial and temperal patterns of mesozooplankton in the Arabian Sea influenced by seasons, upwelling, and oxygen concentrations [24]. And the study on the influence of a tropical cyclone on Chl-a concentration has been executed in the Arabian Sea [25]. However, responses of phytoplankton to strong cyclones in the Arabian Sea were seldom investigated in previous studies.

In this study, we focused on the influence of Hurricane Gonu on phytoplankton in the northern Arabian Sea. The two main issues of our concerns are what happened to the phytoplankton after Hurricane Gonu's passage and what processes were involved. We will present Gonu-induced phytoplankton blooms in the region, and discuss the mechanisms behind these blooms.

Our preliminary research showed that phytoplankton biomass increased several days after Hurricane Gonu's passage, with higher Chl-a concentration compared with that before the storm and with the same period in other years when storms were absent. To explore the ocean responses to Hurricane Gonu, Microwave SST, QuikSCAT wind and Ekman pumping velocity (EPV) were employed to reflect the physical responses during and after Hurricane Gonu's passage; characteristics of upper-ocean stratifications during pre-Gonu and post-Gonu periods were also examined. The EPV associated with wind-stress variability was calculated according to Zhao and Tang [26].

## 2. Data and Methodology

### 2.1. Satellite Products and Hurricane Data

The following products based on satellite measurements were used in this study. The combined product of daily SST (TMI_AMSRE) was obtained based on the Tropical Rain Measuring Mission (TRMM) Microwave Imager (TMI) and the Advanced Microwave Scanning Radiometer-EOS (AMSR-E). It has a resolution of 0.25° by 0.25° [27]. Due to the cloud-penetrating capacity of both TMI and AMSR-E, the two measurements together can overcome influence of cloudy conditions [27]. Therefore, TMI_AMSRE can provide continuous SST observations with a better resolution before, during, and after a hurricane, which is particularly useful during a hurricane. The surface wind data are based on the microwave scatterometer SeaWinds on QuikSCAT satellite that measures surface wind over the oceans [28]. The daily QuikSCAT data [28] downloaded from http://poet.jpl.nasa.gov were used to study Hurricane Gonu.

Merged Aqua/SeaWiFS-derived Chl-a product with 9-km resolution was obtained from the Distributed Active Archive Center (DAAC) of National Aeronautics and Space Administration (NASA; ftp://oceans.gsfc.nasa.gov/Merged/). Both monthly and 8-day Chl-a data were used in the present study. In order to expatiate quantitatively on the relationship between Chl-a concentration and oceanic conditions (including surface winds), we chose two boxes, Box A (65-69°E, 16-19°N) and Box B (59.5-63.5°E, 20.5-23.5°N) in Figure 1 for time series during Gonu, where the variations of wind speed, SST, and EPV were more notable. Considering the lag time of phytoplankton growth for uptake of nutrients (the average turnover time being 2-6 days [29]) and advections by the summer anti-cyclone circulation in the area [23, 30], one patch of high Chl-a concentration moved northward in the western Arabian Sea and another moved southward in the eastern Arabian Sea. Thus, we chose Box A1 (67.5-70.5°E, 12.4-17.2°N) and Box B1 (60-63°E, 21-22.8°N) in Figure 6a as sampling areas for Chl-a.

SeaWiFS-derived total suspended sediment (TSS) as a proxy for determining the influence from terrestrial materials was produced and analyzed. From the daily SeaWiFS files (available at http://oceancolor.gsfc.nasa.gov/cgi), the nLw (normalized water-leaving radiance) 443 and 555 products were extracted. This was followed by the derivation of the daily composites of the two bands. TSS calculation was then made by utilizing the daily images of the two bands and applying

(1) $$\text{TSS}=3.2602\times {\left({\text{R}}_{443}/{\text{R}}_{555}\right)}^{‐3.9322}$$

which was described by Pan *et al*. [31]. Here, R_443_ and R_555_ represent the nLw 443 nm and 551 nm, respectively. Furthermore, the daily TSS images were processed into mean images to discuss variations of TSS during pre-Gonu and post-Gonu periods.

The hurricane data used in this study were downloaded from the Unisys Weather website (http://weather.unisys.com/hurricane/n_indian/), which is based on the best hurricane-track data from the Joint Typhoon Warning Center of USA. The data include the maximum sustained surface wind and the longitude and latitude of the hurricane center every 6 hours. The translation speed (i.e. speed of movement) of a hurricane was thus estimated based on the 6-hourly position of its center in our analysis. And the maximum sustained surface wind speeds were labeled for the period of Hurricane Gonu (Figure 1).

### 2.2. Hydrographic Data

The hydrographic profiles are available from the National Oceanographic Data Center (NODC) of National Oceanic and Atmospheric Administration [32]. Conductivity-Temperature-Depth (CTD) data were downloaded from http://www.aoml.noaa.gov. We used only the CTD data at station Q2900769 (67.78°E, 18.54°N) inside Box A. Vertical profiles of SST and salinity were used to show the influence of Hurricane Gonu on the upper ocean.

### 2.3 Methodology

#### Sea Surface Wind Vectors (SSWVs)

In order to analyze the wind variation before and during Gonu, daily SSWVs were averaged over May 15-31and June 2-6, 2007, respectively. Time series of wind speeds were obtained by averaging daily data over Boxes A and B.

#### Ekman Pumping Velocity (EPV)

EPV is an important index for upwelling in the ocean. It can help to understand the variation of phytoplankton in the study area. EPV was estimated from the QuikSCAT data using the equations in [33] and [26].

#### Mixed Layer Depth (MLD)

We define the MLD as the depth at which temperature is decreased by 1°C from its average in the top 10 m, as in [34]. The MLD was estimated based on the CTD data.

#### Chl-a Concentrations

Chl-a concentrations were first averaged over two periods: the pre-Gonu period (May 15-31, 2007), and the post-Gonu period (20 days after Hurricane Gonu's passage, i.e., June 7-26, 2007). The post-Gonu period was so chosen because phytoplankton blooms generally appear several days after a storm, last 3∼4 weeks, and decay to the nominal pre-storm level after about one month and because there were relatively sparse valid observations of Chl-a due to cloudy weather conditions [2-4].

#### Time Series of Satellite Data

To investigate the temporal evolution of ocean responses to Gonu's passage, time series of wind speed, EPV, and SST were averaged over Boxes A and B (Figure 1), where the changes were more evident. To address the spatial and temporal variations, the Chl-a time series was sampled over Boxes A1 and B1 (Figure 6a). Note that the two boxes used for Chl-a time series were not coincident with the boxes for the other time series; this is because the locations of the phytoplankton blooms were displaced by the summer anti-cyclone circulation.

## 3. Results

### 3.1. Hurricane Gonu Viewed by QuikSCAT Sensor

Hurricane Gonu originated from a tropical storm with a maximum sustained wind (MSW) of 21 m·s^-1^ near 67.7°E and 15.1°N in the southeastern Arabian Sea at 00:00 UTC on June 2, 2007, and rapidly intensified to a hurricane with a MSW up to 72 m s^-1^ at 12:00 UTC on June 4 (Figure 1). The MSW decayed gradually to 18 m s^-1^ at 0600UTC on June 7 when it made a landfall in southern Iran. It was one of the strongest cyclones, with Category 5, i.e., a super cyclone. The translation speed of Hurricane Gonu was slow on the first day of becoming a tropical storm, reached the maximum of 4.9 m s^-1^ at 06:00UTC on June 4 and reduced approximately to 1.6 m s^-1^ during its last days, with a mean translation speed of 3.9 m s^-1^ (Table 1). Gonu's intensifying tendency was similar to that of the translation speed (Table 1; Figure 1). Comparing the intensity and translation speed during Gonu's passage, the translation speed (3.2 m s^-1^) from 18:00UTC on June 4 to 12:00 UTC on June 6, i.e. from the location at 63.2°E, 20.5°N to the location at 59.5°E, 23.1°N (Box B in Figure 1), was slower than that (3.4 m s^-1^) for Box A before 18:00UTC on June 4 under a similar intensity (56 m s^-1^ vs. 55 m s^-1^). And the mean translation speed reached the maximum of 4.6 m s^-1^ when the wind speed of Hurricane Gonu was the strongest (the mean MSW of 61 m s^-1^; the grey oval region in Figure 1).

**Figure 2.**
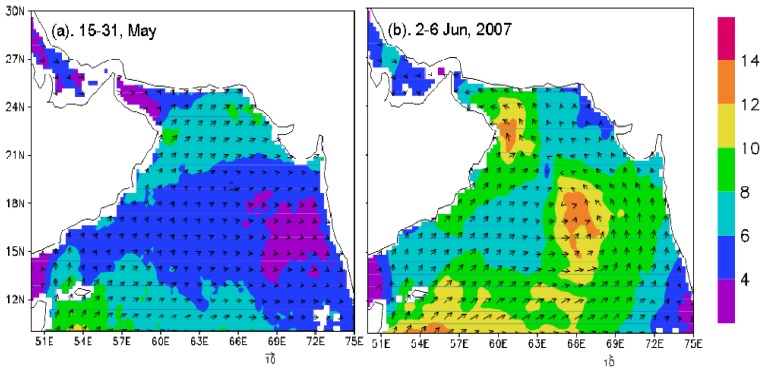
Wind vectors (m s^-1^) before and during Gonu. (a) Winds averaged for the pre-Gonu period of May 15-31, 2007; (b) winds averaged for the period of June 2-6, 2007.

During the pre-Gonu period, the wind speed in the Arabian Sea (including the Gulf of Oman) was generally weak (<8 m s^-1^), and even lower in the central, eastern, and northwestern Arabian Sea (Figure 2a). The wind speed averaged over the Gonu period was evidently stronger (> 8 m s^-1^; Figure 2b) than that of the pre-Gonu period over most of the Arabian Sea, especially over the eastern central and northwestern areas (> 12 m s^-1^; Boxes A and B in Figure 1).

### 3.2. Oceanic Responses

#### SST

**Figure 3.**
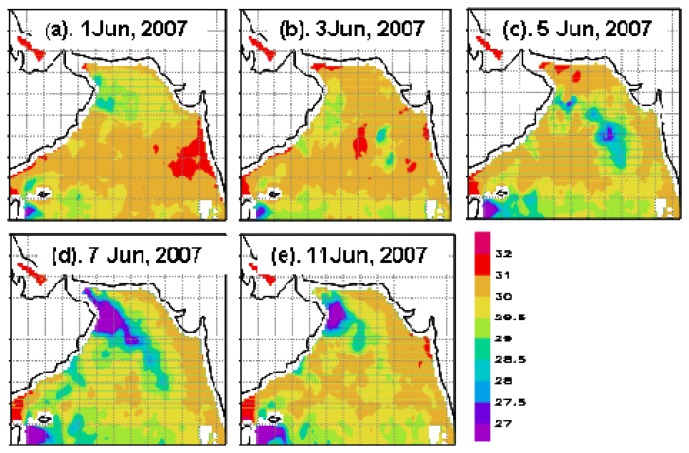
Daily SST (°C) images. (a) Pre-Gonu (June 1, 2007); (b-c) during Gonu; (d-e) post-Gonu.

The daily TMI_AMSRE SST image on June 1, 2007 was chosen as the representative for pre-Gonu ocean state displaying prevalently high SST (>29.5 °C) over most of the Arabian Sea (Figure 3a). During and after Gonu, SST fell over the entire Arabian Sea, and there were two low-SST patches, roughly coincided with the areas of strong winds in Figure 2b: one (<28°C; Figs. 3b-3e) located in the eastern central Arabian Sea (near 67°E, 18°N), which appeared on June 3, was intensified during June 4-5 and decayed on June 7; the other (<27.5°C; Figs. 3c-3e) was more noticeable in the northeastern Arabian Sea, which emerged on June 4, and expanded and intensified gradually for at least 10 days.

#### EPV

**Figure 4.**
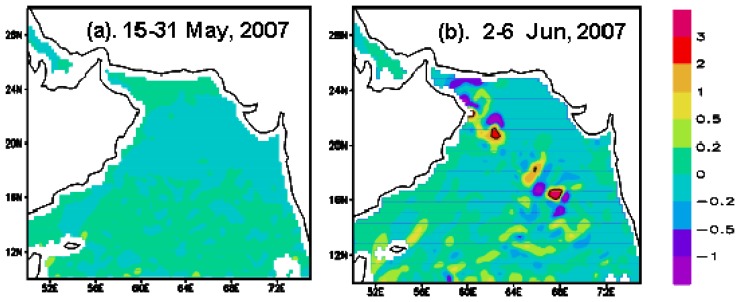
Average Ekman pumping velocity (EPV; positive values pointing upward in 10^-4^ m s^-1^). (a) Pre-Gonu (May 15-31, 2007); (b) during Gonu (June 2–6, 2007).

The EPVs derived from wind stress revealed the upwelling conditions before and after Gonu (Figs. 4a-4b). The pre-Gonu EPV was generally small (< 0.1×10^-4^ m s^-1^; Figure 4a), coincided with low wind speeds during that period, and indicated generally weak upwelling or downwelling in the Arabian Sea. The EPV averaged for the Gonu period displayed strong upwelling and downwelling (magnitude larger than 0.15×10^-4^ m s^-1^) near the hurricane track, up to 0.2×10^-3^ m s^-1^ in Boxes A and B (Figure 4b).

#### Vertical Profiles of Temperature and Salinity

**Figure 5.**
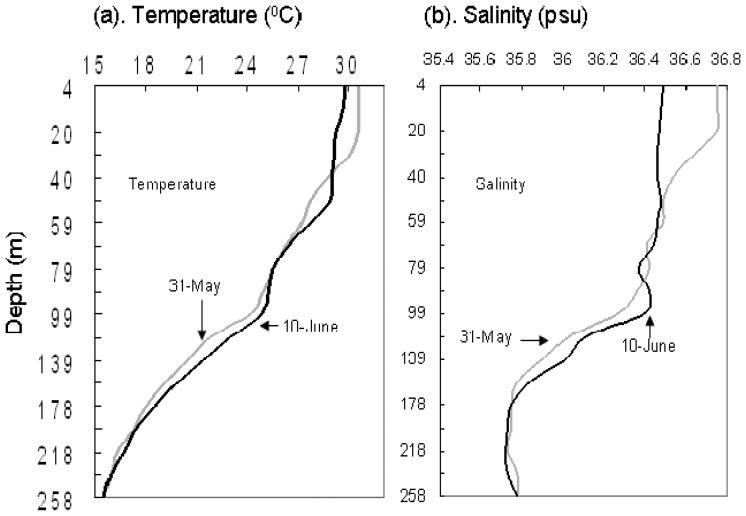
Vertical profiles of temperature and salinity before and after Gonu at (67.78°E, 18.54°N) inside of Box A in Figure 1.

The temperature and salinity profiles showed clear changes before and after Gonu (Figure 5). The MLD was about 31 m on May 31 with high SST of 30.58°C; after Gonu, it deepened to 52 m on June 10 with low SST of 29.7°C (Figure 5a). The depth of halocline was about 20 m before Gonu and reached 90 m after Gonu (Figure 5b). Vertically, the variation of salinity was less than 0.15 psu over a 90-m depth range after Gonu, while the variation before Gonu was over 0.20 psu over a depth of 40 m (Figure 5b).

#### Phytoplankton and TSS

**Figure 6.**
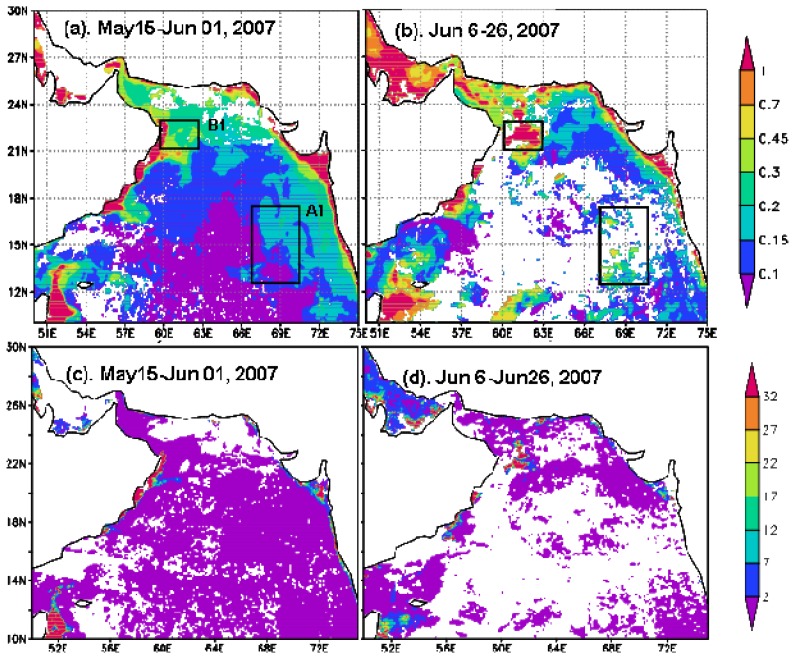
Chl-*a* images in the Arabian Sea (mg m^-3^). (a) Pre-Gonu (averaged over May 15-June 1, 2007); (b) Post-Gonu (averaged over June 7-26). Boxes A1 and B1 in (a) were Chl-*a* sampling areas, where high Chl-*a* concentrations were more evident after Gonu.

**Figure 7.**
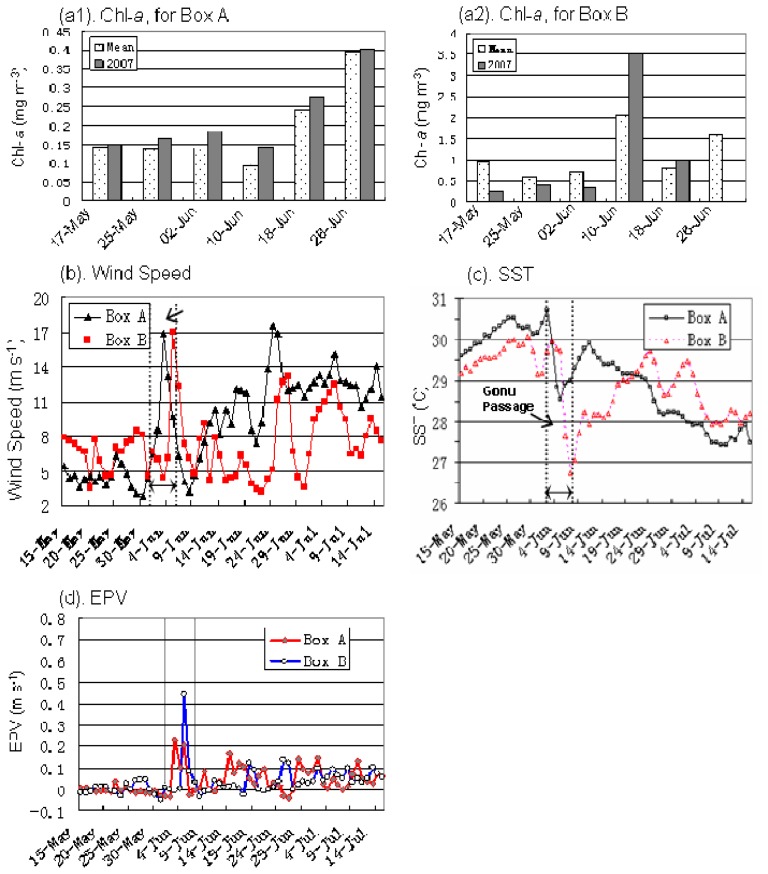
Time series of various data sets. (a) The 8-day-mean Chl-*a* (mg m^-3^) for Boxes A1 and B2 in Figure 6a. The date ticks along the x-axis indicated the first day of the corresponding 8 days). (b) Wind speed (m s^-1^). (c) SST (°C). (d) Ekman pumping velocity (EPV; 10^-4^ m s^-1^) averaged over Boxes A and B during May 15–July 15, 2007.

Chl-*a* displayed a great change before and after Gonu (Figs. 6a-6b and 7a). The pre-Gonu Chl-*a* showed a typical summer oligotrophic condition, with low Chl-*a*, predominantly less than 0.2 mg m^-3^ in the offshore area with water depth over 2,000 m in the northern Arabian Sea (Figure 6a). There was even lower Chl-*a* concentration (< 0.1 mg m^-3^) during the pre-Gonu period in the central Arabian Sea (Figure 6b). After Gonu, Chl-*a* was evidently higher in most of the Arabian Sea, especially in the two boxes in Figure 6b (> 0.3 mg m^-3^). The two high Chl-*a* patches were located in the eastern central Arabian Sea (Box A1 in Figure 6a; > 0.25 mg m^-3^) and northeast of Oman (Box B1 in Fig 6a; > 1 mg m^-3^). There was no evidence for high Chl-*a* in the region with the strongest wind speed of Gonu (the grey oval region in Figure 1).

The TSS images displayed similar distribution with low concentration (<2 g m^-3^) in most of offshore regions before and after Gonu (Figs. 6c-6d). However, the post-Gonu TSS (Figure 6d) increased 5-10 times than that in the pre-Gonu in the offshore area in the northwestern Arabian Sea (Figure 6c), coinciding with the post-Gonu patch of high Chl-*a* (Box B in Figure 6b). And no evident changes were observed in Box A during pre-Gonu and post-Gonu periods.

The time series of Chl-*a* for Box A1 (using 8-day averages) showed that Chl-*a* increased rapidly from 0.16 mg m^-3^ to 0.185 mg m^-3^ in one week after Gonu (Figure 7a1). The increasing tendency of Chl-*a* concentration from May-June can be observed from the mean Chl-*a* data averaged for 2003-2006 and for 2007 Chl-*a* data (Figure 7a1), and is especially obvious in 2007. The time series for Box B1 (Figure 7a2) increased from 0.34 mg m^-3^ to 3.5 mg m^-3^ in the first week after Gonu, with about 5-8 days lag. And Chl-*a* was generally higher in concentration for June than that for May, especially higher in the second week of June, whether in the mean or in 2007 Chl-*a* time series.

## 4. Discussions

It is well-known that nutrients are one of the most important factors that can limit phytoplankton growth in euphotic tropical oceans. Available nutrients in the summer euphotic layer are generally poor due to the consumption of phytoplankton photosynthesis in the light-rich tropical oceans. However, absent of solar radiation, phytoplankton growth cannot ocurr in deep water columns, so there are generally abundant nutrients below the euphotic layer [19; 35-36]. Here, we studied the influence of oceanographic conditions on phytoplankton biomass by investigating weather conditions, upwelling, mixing, SST, and distributions of temperature and salinity in the water column.

### 4.1. Overall Increase of Phytoplankton in the Arabian Sea

The surface Chl-*a* in the Arabian Sea displayed a great temporal-spatial variation during pre-Gonu and post-Gonu periods. The regions of great variation were mainly located in offshore areas, where the water depth is over 1,000 m and the distance is over 50 km away from the coastline; therefore, the effect of re-suspension from bottom materials and riverine input on Chl-*a* is limited in these regions.

In tropical oligotrophic regions, the availability of nutrients is the most important factor in limiting the growth of phytoplankton. During the pre-Gonu period, high SST (29-30.8°C), weak wind speed (2-8.2 m s^-1^), and weak EPV (< 0.1×10^-4^ m s^-1^) were prevailing in the entire Arabian Sea. High SST generally enhances stratification of the upper ocean; weak wind produces weak vertical mixing and weak EPV indicated weak upwelling of subsurface water. These unfavorable conditions of entrainment mixing and upwelling implied weak supply of nutrients during the pre-Gonu period. In addition, the vertical profiles of temperature and salinity indicated also that the MLD was very shallow during this period. These processes accounted for the low Chl-*a* pattern (0-0.2 mg m^-3^) during the pre-Gonu period.

During Hurricane Gonu, significantly stronger winds (> 8 m s^-1^) were observed over the entire Arabian Sea, with the strongest (> 20 m s^-1^) near the Hurricane Gonu's track (Figure 2b), compared with generally very weak winds (< 7 m s^-1^; Figure 2a) during the pre-Gonu period. In addition, stronger upwelling (> 0.15×10^-4^ m s^-1^) was seen during Hurricane Gonu, especially in Boxes A and B (Figure 4b) where the maximum EPV was greater than 0.2×10^-3^ m s^-1^, roughly matching the two high Chl-*a* patches in Figure 6b and strong wind regions in Figure 2b. In contrast, very weak upwelling (< 0.1×10^-4^ m s^-1^) existed during the pre-Gonu period. The SST dropped over most of the Arabian Sea during and after Gonu, especially in Boxes A and B (Figure 3d), coinciding with the two high Chl-*a* patches, as well as with the two strong wind and upwelling locations. Therefore, strong winds and upwelling together with decreased SST led to supply of nutrients from beneath the euphotic layer, inducing extensively increase of phytoplankton. And temperature and salinity in the upper ocean showed a deeper MLD compared with the pre-Gonu period; it also showed smaller salinity gradient during post-Gonu period (Figure 5), affirming the effect of stronger mixing, upwelling, and entrainment of nutrients induced by Gonu.

**Figure 8.**
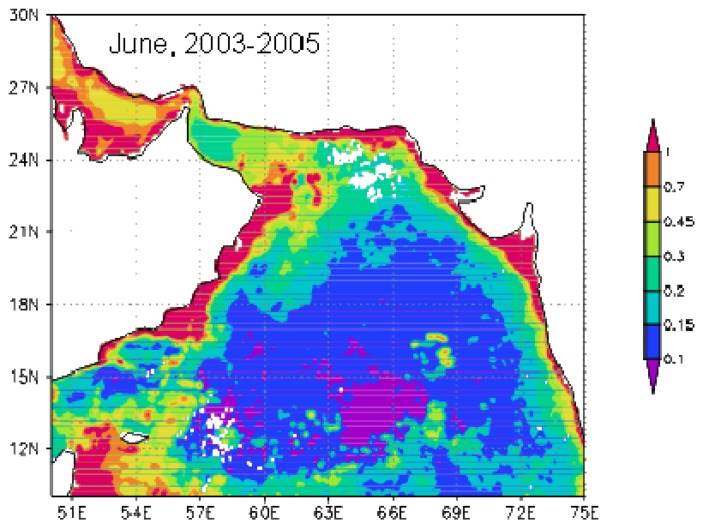
Chl-*a* image for June averaged from 2003-2005 in the Arabian Sea (mg m^-3^).

Due to seasonal increasing tendency of phytoplankton biomass from late spring to early summer [19-21], the mean Chl-*a* image for June from 2003 to 2005 (Figure 8) was produced in order to analyze the influence of southwest monsoon. The 3-yr-mean Chl-*a* for June (Figure 8) displayed higher concentration than that for May, 2007 (Fig 6a) but lower concentration than that for June, 2007 (Fig 6b) in most of the Arabian Sea, especially in the central Arabian Sea (Box A) and off Oman (Box B). Although summer monsoon enhances phytoplankton in June through turbulent entrainment, offshore transport of coastal upwelled water, and mesoscale eddies, the results also affirmed strong influence of the storm on phytoplankton in the Arabian Sea.

TSS images displayed roughly similar spatial pattern with low TSS concentrations in most of offshore Arabian Sea, but high TSS concentration was observed off Oman after Gonu, with 4-10 times' increase compared with that before Gonu, where the coastal upwelling/dust storms occurs also in summer through offshore Ekman pumping transport or wind [9, 11, 23]. Thus, terrestrial input may imply offshore supply of dust storms as well as nutrient uptake from deeper subsurface waters, triggering also high phytoplankton biomass in the regions off Oman.

### 4.2. Different Intensities of the Two Chl-a Blooms

In order to quantitatively discuss the influence of Gonu on the two high Chl-*a* patches, the time series of oceanic conditions and Chl-*a* were produced before, during, and after Gonu (Figs. 7a, 7b, 7c and 7d). Before Gonu, there were generally low Chl-*a* concentrations (Figure 7a), associated with weak winds (Figure 7b), high SST (Figure 7c), and weak EPV (Figure 7d). The wind speed during Gonu was up to 17 m s^-1^, 2∼4 times larger than the pre-Gonu wind speed. The wind speeds during Hurricane Gonu were roughly equivalent over both Boxes A and B. Although the EPV (Figure 7d) displayed stronger upwelling tendency (> 0.2×10^-4^ m s^-1^) for the two regions during Hurricane Gonu, the upwelling tendency for Box B was more obvious than that for Box A. SST began to fall after Gonu's passage, in response to increasing winds and EPV associated with Hurricane Gonu; however, the decreasing SST in Box B was more evident than that in Box A. The time series (Figure 8a) showed that the Chl-*a* in Box A1 increased rapidly from the pre-hurricane 0.16 mg m^-3^ to 0.185 mg m^-3^ in the first week after Gonu,4-day lag (Figure 7a1) with about 42% increase from June 2 to June 17 in total compared with the mean Chl-*a* for 2003-2005; The time series for Box B1 (Figure 7a2) went from 0.34 mg m^-3^ to 3.5 mg m^-3^ in one week after Gonu, with about 46% increase than the mean Chl-*a* concentration. The results implied the influence of Gonu on increase of Chl-*a* and the more notable tendency of Chl-*a* increase for Box B1 than for Box A1.

Previous studies reported high Chl-*a* patches produced by moderate hurricanes/cyclones, where translation speeds of the two storms were the slowest during their strongest intensities [2; 5]. Thus, it was difficult to estimate which factor, the translation speed or the intensity, exerted more influence on phytoplankton blooms. In the present study, the mean translation speed during the strongest period of Gonu reached up to 4.9 m s^-1^, thus the relatively short lingering time shortened the influence of entrainment/upwelling and mixing induced by Hurricane Gonu; as a result, the Chl-*a* increase in the region with the strongest wind speed of Hurricane Gonu was not more obvious than that in the two high Chl-*a* boxes (Figure 1) with relatively slow translation speed. Under similar speeds, the mean translation speed during Hurricane Gonu was lower over Box B (∼3.2 m s^-1^) with stronger upwelling tendency than that over Box A (3.4 m s^-1^) with weaker upwelling tendency. For cyclones with similar intensities, a slower translation speed (in other words, longer lingering time) exerted much longer influence on upper oceans, possibly enhancing tendency of entrainment mixing and upwelling caused by cyclones, which induces more injection of nutrients from the subsurface water. According to [7], for similar cyclones with nondimensional storm speeds near unity, inertial resonance may have evidently contributed to the Chl-*a* responses for longer lingering cyclones. Thus, these may lead to more evident change of Chl-*a* in Box B1 (mean moving speed: 3.2 m s^-1^) than that in Box A1 (mean moving speed: 3.4 m s^-1^) after Hurricane Gonu. In addition, the SST decrease could signal upwelling of cold water from the subsurface layer and the SST recovery could indicate a re-stratification of the upper ocean, which can influence the entrainment of nutrient-rich subsurface water [37]. Moreover, the low SST in Box A disappeared more rapidly than that in Box B, which is consistent with the fact that the Chl-*a* bloom in Box B1 was more noticeable among the two high Chl-*a* patches. These implied that, for super cyclones like Hurricane Gonu, effect of their lingering time on phytoplankton seemed as important as their intensities, due to longer action time of upwelling and mixing produced by the storms.

Evidence from TSS (Figure 8) displayed that high suspended material concentration appeared in the region of Box B off Oman, where coastal upwelling and Ekman offshore transport prevails in summer season [9; 11]. Excessive terrestrial input may also intensify the bloom patch off Oman to some degree. And compared with the biological response within Box A in oligotrophic central Arabian Sea, moderate perturbation can reach a significant nutrient reservoir within Box B off Oman where there is a significant nutrient reservoir [22]. Thus, although we affirmed that Gonu induced the two high Chl-*a* patches, influence of terrestrial materials, locations and input from Ekman offshore transport on increase of Chl-a concentration may still be probed further for Box B off Oman after Gonu. In future study, we will pay more attention to the influence of terrestrial inputs (such as dust storms) and Ekman offshore transport from coastal upwelling on Chl-*a* concentration off Oman in summer.

## 5. Summary

The present study suggested that the variation of Chl-*a* in the Arabian Sea before and after a hurricane was related to strong storm-induced mixing/upwelling. Chl-*a* concentrations increased generally over most of the Arabian Sea, especially along the hurricane track. The significant increases of Chl-*a* concentrations (i.e. the two high Chl-*a* patches) were located where the translation speeds were slow under the moderate strong wind speed of the hurricane. For super cyclones like Gonu, effect of their lingering time on phytoplankton seemed as important as that of their intensities, due to longer action time of upwelling and mixing/inertial resonances produced by the storms.

## Figures and Tables

**Table 1.** Maximum sustained wind and translation speed during Gonu's passage (m s-1).

**Parameter**	**The whole passage**	**Box A**	**Box B**
Mean moving speed*	3.9	3.4	3.2
Range of moving speed*	2.1-4.9	2.7-4.4	2. 2-3.8
MSW	72	67	72
Mean MSW	61	55	56

MSW: Maximum Sustained Wind. Units: m s^-1^.

* Estimation for value derived from cyclones data of Unisys Weather exclusive of the data during a tropical storm (MSW< 34 m s^-1^)
